# Transcriptomic Analysis of *Betula halophila* in Response to Salt Stress

**DOI:** 10.3390/ijms19113412

**Published:** 2018-10-31

**Authors:** Fenjuan Shao, Lisha Zhang, Iain W. Wilson, Deyou Qiu

**Affiliations:** 1 State Key Laboratory of Tree Genetics and Breeding, Key Laboratory of Tree Breeding and Cultivation of State Forestry Administration, The Research Institute of Forestry, Chinese Academy of Forestry, Beijing 100091, China; shaofenjuan@caf.ac.cn (F.S.); zlsxxb@aliyun.com (L.Z.); 2 CSIRO Agriculture and Food, Canberra, ACT 2601, Australia; Iain.Wilson@csiro.au

**Keywords:** *Betula halophile*, salt stress, transcriptomes

## Abstract

Soil salinization is a matter of concern worldwide. It can eventually lead to the desertification of land and severely damage local agricultural production and the ecological environment. *Betula halophila* is a tree with high salt tolerance, so it is of importance to understand and discover the salt responsive genes of *B. halophila* for breeding salinity resistant varieties of trees. However, there is no report on the transcriptome in response to salt stress in *B. halophila*. Using Illumina sequencing platform, approximately 460 M raw reads were generated and assembled into 117,091 unigenes. Among these unigenes, 64,551 unigenes (55.12%) were annotated with gene descriptions, while the other 44.88% were unknown. 168 up-regulated genes and 351 down-regulated genes were identified, respectively. These Differentially Expressed Genes (DEGs) involved in multiple pathways including the Salt Overly Sensitive (SOS) pathway, ion transport and uptake, antioxidant enzyme, ABA signal pathway and so on. The gene ontology (GO) enrichments suggested that the DEGs were mainly involved in a plant-type cell wall organization biological process, cell wall cellular component, and structural constituent of cell wall molecular function. Kyoto Encyclopedia of Genes and Genomes (KEGG) pathway enrichment showed that the top-four enriched pathways were ‘Fatty acid elongation’, ‘Ribosome’, ‘Sphingolipid metabolism’ and ‘Flavonoid biosynthesis’. The expression patterns of sixteen DEGs were analyzed by qRT-PCR to verify the RNA-seq data. Among them, the transcription factor AT-Hook Motif Nuclear Localized gene and dehydrins might play an important role in response to salt stress in *B. halophila*. Our results provide an important gene resource to breed salt tolerant plants and useful information for further elucidation of the molecular mechanism of salt tolerance in *B. halophila*.

## 1. Introduction

Soil salinization is worldwide problem that can alter the soil osmotic potential to the point where it inhibits the uptake of water by plants, severely impacting agricultural production and the ecological environment. It has been reported that more than 6% of the world’s land is affected by salt [1,2,3], and increased salinization may lead to the loss of 30% arable land in the next 25 years. It has been reported that more than 6% of the world’s land is affected by salt [1,2,3], and increased salinization may lead to the loss of 30% arable land in the next 25 years and up to 50% by 2050 [1,2,3]. Therefore, soil salinization is a serious threat to the growth and development of plants. At present, it is particularly urgent to search for salinity resistant varieties of plants and screen for salt tolerant gene alleles or transform them genetically to enable plants to grow and reproduce with increasing salinity stress [4]. Moreover, understanding the mechanism of salt tolerance in plants can provide valuable information for effective engineering strategies.

In plants, the salt resistance mechanism is very complicated and involves a complex of processes at the molecular, cellular, metabolic, physiological, and whole-plant levels. Once the plant is under salt stress, multiple signal transduction pathways are activated to cope with salt stress [2,4,5,6]. In recent years, although extensive studies among ion uptake and transport, osmotic regulation, hormone metabolism, antioxidant metabolism, and stress signaling have made significant progress [4,5,6,7,8,9,10,11], the molecular mechanisms involved in salt tolerance remain to be elucidated. In addition, next-generation high-throughput sequencing based RNA-seq analysis has been widely used to uncover expression patterns under abiotic stress, and it provides a comprehensive means of identifying and studying the differential expression genes [12,13,14,15].

*Betula halophila* is a haloduric species in China, belonging to the family Betulaceae. It was first discovered in a swamp with extremely high salinity in Xinjiang province [16] in 1956 by Professor Renchang Qin. *B. halophila* is a critically endangered plant, which has high salt tolerance, and high ecological and economic value in promoting the afforestation of saline soil in arid and semi-arid areas16. Thus *B. halophila* is a potent source of salt tolerant genes. However, to the best of our knowledge, there is no published information on genes associated with salt tolerance in *B. halophila*. Understanding the molecular mechanisms of salt tolerance are potentially important for breeding salt tolerant varieties. With the aim of identifying the genes in response to increasing salt concentration and potentially the molecular mechanisms of salt tolerance in *B. halophila*, we constructed transcriptome libraries from the leaves of control *B. halophila* plants and plants subjected to salt treatment. The aims were to detect salt responsive genes from *B. halophila* and explore their roles in response to salt stress. Our results provide insight into the molecular mechanisms of salt tolerance in *B. halophila*. A better understanding of these tolerance mechanisms can be used to breed crops with improved yield performance under salinity stress.

## 2. Results

### 2.1. Transcriptome Sequencing and Assembly

**Figure 1:**
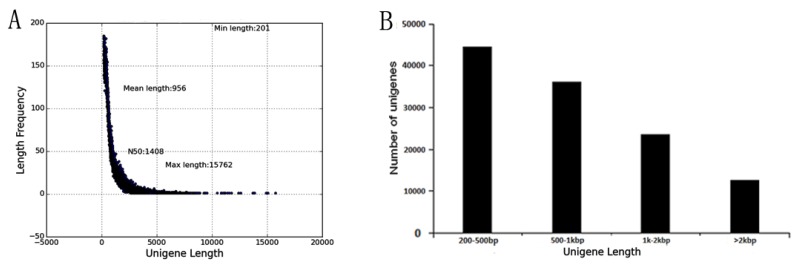
Length distribution of the assembled unigenes. (**A**) The number of contigs with same length. (**B**) The number of contigs with length 200–500 bp, 500 bp−1 kb, 1–2 kb and larger than 2 kb are shown.

In order to explore the salt tolerant genes of *B. halophila*, six cDNA libraries were constructed from leaves of eight-months-old control plants (untreated) and plants treated with 200 mM of NaCl for 24 h, and sequenced using the Illumina deep sequencing platform. A total of 68,348,352, 75,684,144, 70,510,750, 86,101,536, 77,402,824, and 84,837,142 raw reads were generated by Illumina sequencing, respectively, after adapter sequence and low quality sequences had been removed, a total of 66,834,236, 73,359,338, 69,082,978, 84,155,542, 75,577,004, and 82,931,756 clean reads were obtained, and the Q20 percentage (proportion of nucleotides with a quality value larger than 20) for each data was 96.5%, 96.48%, 96.25%, 96.54%, 96.49%, and 96.45%, respectively. The GC (%) ratio for each library was 46.88%, 47.4%, 47.29%, 47.28%, 47.33% and 47.29% (Table 1). Transcriptome assembly was accomplished based on the left.fq and right.fq using Trinity with the min_kmer_cov set to 2 by default and all other parameters set to default [17]. As a result, a total of 117,091 unigenes with lengths ranging from 201 bp to 15,762 bp were obtained. The size distribution of the unigenes is shown in Figure 1. The size distribution showed that the unigenes ranged from 200 bp to 1 kbp was the majority (Figure 1). The average length, median length, and N50 of the assembled unigenes were 956 bp, 631 bp and 1408 bp, separately. The total length of 117,091 unigenes was 110 Mb, which suggests that most of the sequencing data had been successfully assembled into relatively long unigenes.

### 2.2. Functional Annotation and Classification of the Unigenes

Functional annotation of unigenes were performed to search for homologues against the NCBI non-redundant protein sequence database (*N*r), NCBI nucleotide sequences (*N*t), Pfam (Protein family), Kyoto Encyclopedia of Genes and Genomes (KEGG), swiss-prot sequence databases (SwissProt), Gene ontology (GO), and Eukaryotic Orthologous Groups (KOG) using the Basic Local Alignment Search Tool (BLAST) [18]. An e-value cut-off of 10^−5^ was applied to the homologue recognition. The results were shown in Table 2. 64551 (55.12%) total unigenes were annotated in at least one database and 8973 unigenes (7.66%) were annotated in all databases. 51,105 (43.64%) total unigenes were annotated in the *N*r protein database.

**Figure 2:**
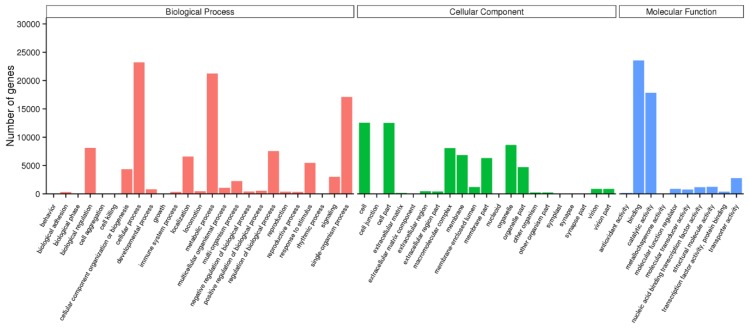
Gene ontology (GO) classification of unigenes. The GO terms are summarized into three main categories: biological process, cellular component, and molecular function.

The GO analysis indicated that a total of 41,116 unigenes were summarized into the three main GO categories (biological process, cellular component, and molecular function) and 56 sub-categories (Figure 2). In the biological process category, genes involved in cellular process, metabolic process, and single-organism process were dominant. As for the cellular component category, genes involved in cell, cell part, and organelle were highly represented. The molecular function category mainly included genes involved in binding and catalytic activity.

**Figure 3:**
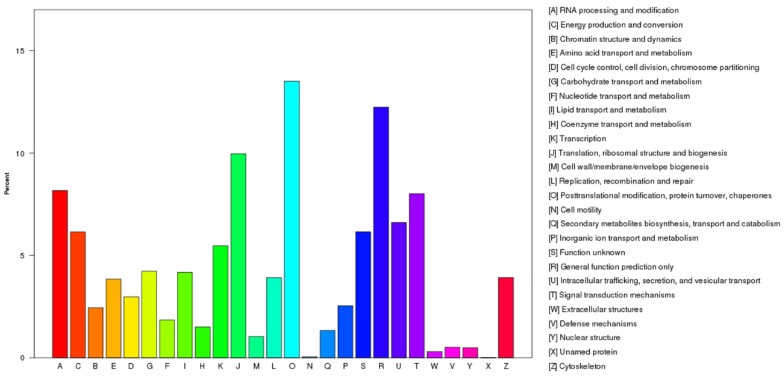
Eukaryotic Orthologous Groups (KOG) classification of the unigenes.

The KOG analysis showed that all of the 15,572 unigenes were divided into 26 different functional classes, which were represented by A to Z (Figure 3). Among the 26 categories, the largest group was ‘Post-translational modification, protein turnover, chaperon’ (2104, 13.51%) followed by ‘General function prediction’ (1906, 12.24%), ‘Translation, ribosomal structure, and biogenesis’ (1552, 9.97%), ‘RNA processing and modification’ (1272, 8.17%) and ‘Signal Transduction’ (1248, 8.01%). The smallest group was ‘Cell motility’ (7, 0.04%) and ‘Unnamed protein’ (2, 0.01%). 

**Figure 4:**
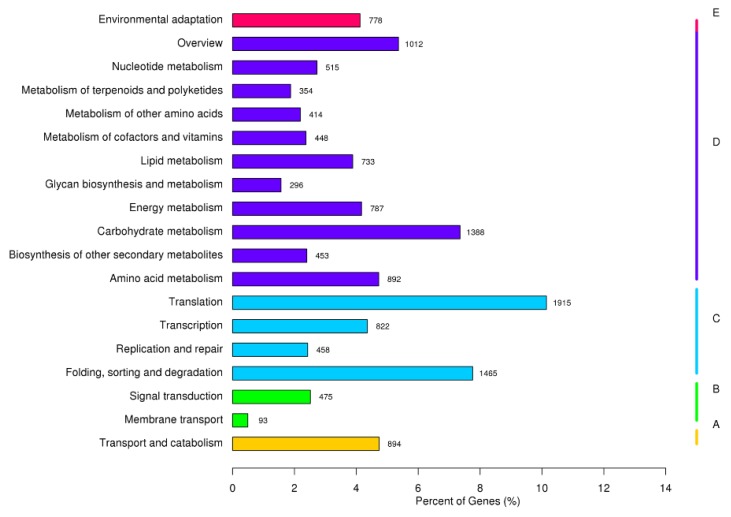
Kyoto Encyclopedia of Genes and Genomes (KEGG) classification of KO annotated unigenes.

The KEGG pathway analysis revealed that 18876 (16.12%) of the unigenes could be mapped to the KEGG database and referred to 129 pathways (Figure 4). The pathway involved the highest number of unigenes was ‘Translation’ (1915, 10.14%), followed by ‘Folding, sorting, and degradation’ (1465, 7.76%), ‘Carbohydrate metabolism’ (1388, 7.35%) and ‘Overview’ (1012, 5.36%). These results are very important for studying the mechanism in *B. halophila* response to salt.

### 2.3. Differential Expression Genes in B. halophila Response to Salt

**Figure 5:**
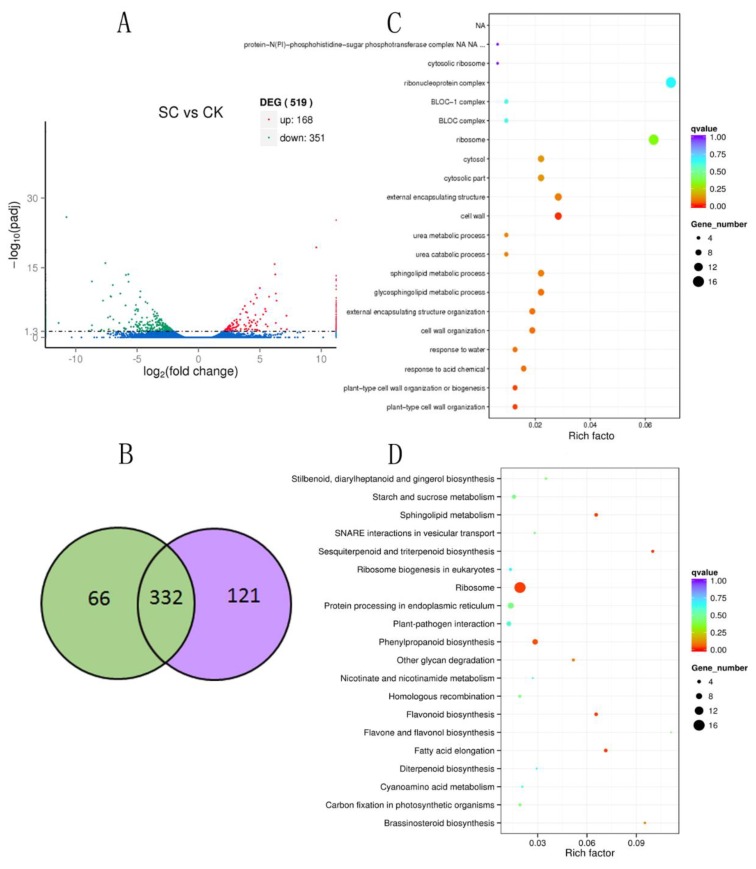
**A**. Up-regulated and down-regulated differentially expressed genes in SC vs. CK; **B**. Venn diagrams showing unique and shared differentially expressed genes (DEGs) in SC (green) vs. CK (purple); **C**. Scatterplot of GO category enrichment of DEGs in SC vs. CK; **D**. Scatterplot of enriched KEGG pathways for DEGs in SC vs. CK. Rich factor is the ratio of the differentially expressed gene number to the total gene number in a certain pathway. The size and color of dot represent the gene number and the range of the q value, respectively.

To obtain the differential expression genes’ response to salt in *B. halophila*, we compared the differentially expressed tags of two libraries. As a results, a total of 519 differentially expressed genes (DEGs) with *q* value < 0.05 and |log2 (fold change)| >1 were identified in the two libraries (Table S1). As shown in Figure 5a, there were more down-regulated genes (351) than up-regulated genes (168). Among these DEGs, 332 DEGs were present in both libraries, (Figure 5b). 66 DEGs were only detected in the salt stress library (Figure 5b) and 121 DEGs were only detected in the control library. In this study, the transcription factor AT-Hook Motif Nuclear Localized gene (AHL) was the most up-regulate gene in leaves after the salt stress. Conversely, a dehydrin (DHNs) was the most down-regulated gene. These results suggest that the two genes may have a high correlation with salt resistance of *B. halophila*. The GO and KEGG classification of the 519 DEGs were analyzed (Figure S1). GO enrichment and KEGG enrichment were performed for further analysis of the functions of 519 DEGs.

### 2.4. GO category Enrichment of DEGs Under Salt Stress

To characterize the function of the DEGs under salt stress, the GO category enrichment analysis was performed using Fisher’s exact test with *p* value ≤0.05 as the cutoff. GO category enrichment analysis for 519 DEGs under salt stress showed that these DEGs were mainly involved in a plant-type cell wall organization biological process, plant-type cell wall organization or biogenesis biological process, cell wall cellular component and structural constituent of cell wall molecular function (Figure 5c, Table S2). For the up-regulated DEGs, metalloendopeptidase activity molecular function was most highly enriched (Table S3). For down-regulated DEGs (Figure 5c), in the BP category, ‘plant-type cell wall organization biological process’, ‘plant-type cell wall organization or biogenesis biological process’, ‘cell wall organization biological process’, and ‘external encapsulating structure organization biological process’ were most highly enriched. In the CC category, ‘cell wall cellular component’, ‘cytosolic part cellular component’, ‘cytosol cellular component’, and ‘external encapsulating structure cellular component’ were the main enriched terms. In MF, the most enriched term was structural constituent of cell wall molecular function (Table S4).

### 2.5. KEGG Enrichment of DEGs under Salt Stress

The KEGG pathway enrichment analysis were performed to identify the candidate pathways involved in salt stress using KOBAS 2.0 [19]. The results showed that genes with KO number within 519 DEGs under salt stress were enriched in 48 KEGG pathways (Table S5). The top-four enriched pathways for DEGs in SC vs CK were ‘Fatty acid elongation’, ‘Ribosome’, ‘Sphingolipid metabolism’ and ‘Flavonoid biosynthesis’. For up-regulated DEGs (Table S6), the most highly enriched pathways were ‘Sphingolipid metabolism’, ‘Other glycan degradation’, ‘Brassinosteroid biosynthesis’ and ‘Citrate cycle (TCA cycle)’ (Figure 5d). For down-regulated DEGs (Table S7), ‘Ribosome’, ‘Fatty acid elongation’, ‘Flavonoid biosynthesis’ and ‘Phenylpropanoid biosynthesis’ were the top-four enriched pathways (Figure 5d).

### 2.6. qRT-PCR Analysis

**Figure 6:**
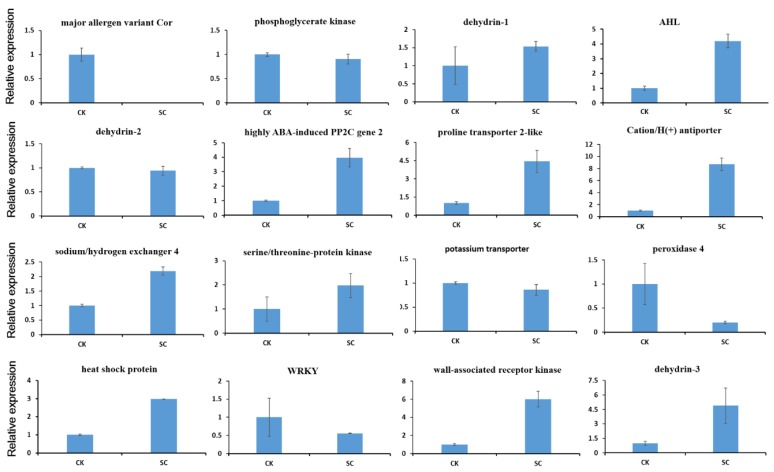
qRT-PCR validation of sixteen selected DEGs in leaves. Fold changes of the DEGs are shown. The expression levels in CK were arbitrarily set to 1. Error bars represent the standard deviations of three technical PCR replicates.

In order to validate the RNA-seq data and confirm the differential expression genes, we performed qRT-PCR on sixteen candidate DEGs associated with salt stress. The results revealed that these DEGs include AHL, dehydrin-1, highly ABA-induced PP2C gene, proline transporter 2-like, sodium/hydrogen exchanger 4, serine/threonine-protein kinase, heat shock protein, Cation/H(+) antiporter, wall-associated receptor kinase-like and dehydrin-3 were up-regulated in the leaves with salt treatment (Figure 6), whereas major allergen variant Cor, dehydrin-2, phosphoglycerate kinase, potassium transporter, peroxidase 4 and WRKY transcription factor were down- regulated in the leaves with salt treatment. The results indicated that these sixteen candidate DEGs had the same expression patterns compared with the sequencing data, suggesting the reliability of the RNA-seq data. 

## 3. Discussion

*B. halophila* is a plant with high salt tolerance, so it is important to discover the salt tolerant genes of *B. halophila* for breeding salinity resistant varieties of trees. However, to our best knowledge, there is no report about genes associated with salt tolerance in *B. halophila*. In this study, we analyzed the transcriptomic data from the leaves of wild type *B. halophila* plants and plants with salt treatment. As a result, approximately 460 M raw reads were generated and were further assembled into 117,091 unigenes, among these unigenes, 64551 unigenes (55%) were annotated with gene descriptions, while the other 45% were unknown. This is the first report of transcriptome data from *B. halophila*. This transcriptome data provides an important genus resource for insight into the molecular mechanism of salt tolerance and facilitates discovery of novel genes responsive to salt stress in *B. halophila.*

In plants, salt stress responsive mechanisms are very complicated, which involve a complex interaction of physiological processes, metabolic pathways, and regulation at the molecular and cellular levels. Although plant response to salt stress has been extensively studied at different levels, the mechanisms underlying salinity tolerance are far from being completely understood. In addition, salt stress responsive mechanisms in different plants are also different. At present, the main mechanisms for which plants respond to salt stress include ion homeostasis and compartmentalization, ion transport and uptake, biosynthesis of osmoprotectants and compatible solutes, activation of antioxidant enzyme, and synthesis of antioxidant compounds [4,5,6,7,11]. In this study, 168 up-regulated genes and 351 down-regulated genes were identified in *B. halophila* under salt stress, respectively. These DEGs include dehydrin proteins, sodium/hydrogen exchanger, potassium transporter, sarcoplasmic/endoplasmic reticulum calcium ATPase, Ca^2+^ antiporter/cation exchanger, Nodulin MtN21/EamA-like transporter, heat shock protein, phosphoenolpyruvatecarboxykinase, NADH dehydrogenase, highly ABA-induced PP2C gene, homeobox-leucine zipper protein, phosphoglycerate kinase, WRKY transcription factor, AP2/ERF and B3 domain-containing transcription factor, flavonoid 3′,5′-hydroxylase, which is consistent with the other plants that are reported to be responsive to salt stress [20,21,22,23,24]. 

The analysis of GO enrichments suggested that the 519 DEGs response to salt stress was mainly involved in plant-type cell wall organization biological process, plant-type cell wall organization or biogenesis biological process, cell wall cellular component and structural constituent of cell wall molecular function. KEGG pathway enrichment results showed that the top-four enriched pathways for DEGs was ‘Fatty acid elongation’, ‘Ribosome’, ‘Sphingolipid metabolism’, and ‘Flavonoid biosynthesis’. The expression patterns of sixteen of these DEGs were analyzed by qRT-PCR to verify the RNA-seq results. It revealed that the qRT-PCR results were consistent with RNA-seq data. 

**Figure 7:**
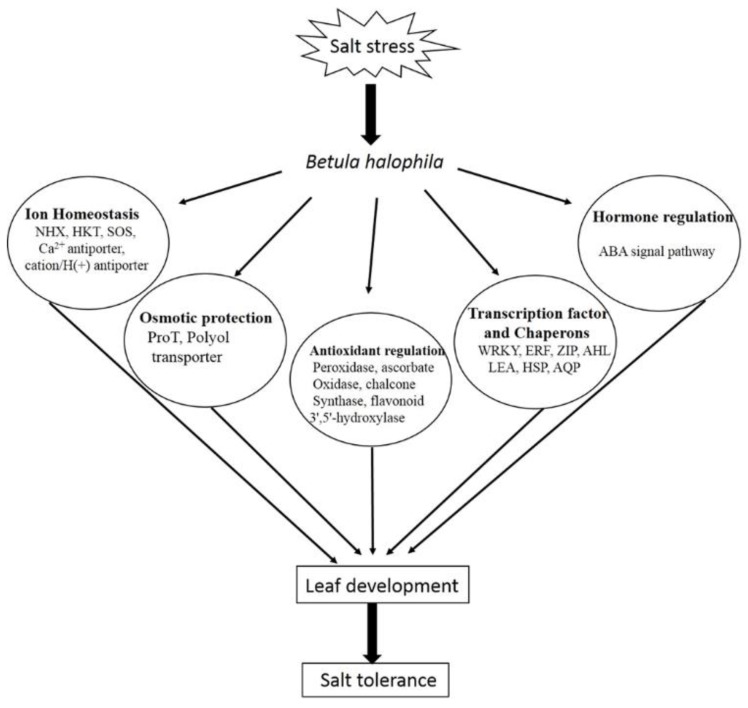
The possible mechanism of salt tolerance in the leaves of *B. halophila.*

Based on the functional annotations of the 519 DEGs and the physiological evidence of *B. halophila* in response to salt stress [25], the possible mechanism of salt tolerance in the leaves of *B. halophila* was summarized in Figure 7. The possible salt tolerance mechanism is coordinately linked with ion homeostasis, osmotic protection, antioxidant regulation, ABA signal pathway, transcription factors and chaperons. When the plant is treatment with 200mM NaCl, multiple signal pathways are activated to cope with salt stress such as the SOS pathway, antioxidant pathway and ABA signal pathway and so on. Meanwhile, the osmoprotectants such as proline and polyols were accumulated to protect the cell. In addition, the transcription factors (WRKY, ERF, ZIP and AHL) and (LEAs, HSPs and AQPs) were activated to regulate the genes involved in the above pathways [2,3,4,5,6]. Overall, the salt tolerance mechanism in *B. halophila* is a complex network that involved the interactions at multiple levels. This information will be useful in elucidating the salt tolerance mechanisms in *B. halophila.*

In the present study, we observed that one dehydrin (DHNs) which is the most down-regulated gene among these DEGs and two other dehydrins showed a distinct salt responsive expression, suggesting that these dehydrin proteins may play different roles in response to salt stress in *B. halophila*. Dehydrins, also known as group 2 LEA (Late Embryogenesis Abundant) proteins, play a fundamental role in plant response to abiotic stresses [26,27,28]. Their expression is often induced under salinity, dehydration, cold and frost stress. Dehydrins are divided into five structural subgroups: Kn, SKn, KnS, YxKn and YxSKn [28]. The three dehydrins protein features of *B. halophila* were all SK3 subclass. It has been shown that SK3 dehydrins play an important protective role in plant stress tolerance, including drought, cold, and salinity [27]. For example, the expression of the durum wheat DHN-5 in A. thaliana led to an increase in salt and osmotic stress tolerance [28]. Rab16A in salt-tolerant Indica rice variety Pokkali can enhance tolerance to drought and salt stress in tobacco plants [29]. Similarly, overexpression of the wheat dehydrin PMA80 (as well as the LEAI protein PMA1959) enhances rice tolerance to drought and salt stress [30]. Although experimental evidence suggests that dehydrins have diverse roles (membrane protection, cryoprotection of enzymes, and protection from reactive oxygen species) in response to stresses [27,28,29,30,31], further efforts are still needed to precisely confirm the roles of these dehydrins and explore the regulatory mechanism underlying these functions in plant adaptive response to abiotic stresses.

In addition, our results indicated that the transcription factor AT-Hook Motif Nuclear Localized gene (AHL) was the most up-regulated gene in leaves after salt stress, implying that it might play an important role in response to salt stress in *B. halophila*. Previous studies showed that the AHL genes regulate diverse aspects of growth and development in plants. Such as the homeostasis of phytohormones [32], and defense responses [33,34,35,36,37,38,39,40]. However, there is no report about the function of AHL genes associated with salt stress. Further studies are still needed to understand the function of AHL genes in salt stress. 

Therefore, our results provide a list of candidate genes for further investigation to determine whether they have a role in allowing *B. halophila* to tolerate high salt levels, and may be helpful in the understanding of the molecular mechanisms of salt stress response in *B. halophila*.

## 4. Materials and Methods

### 4.1. Plant Materials

The seeds of *B. halophila* were obtained from Xinjiang Academy of Forestry. After germination, the seedlings of *B. halophila* were grown in the greenhouse in Chinese Academy of Forestry. Leaves were collected from eight-months-old plants, the fourth or fifth leaf from top to bottom was used for sampling and RNA extraction. Three independent biological replicates were performed for each experiment. All samples were frozen and stored in liquid nitrogen until use.

### 4.2. Salt stress Treatment

Based on *Betula halophila* physiological response to salt stress as described by Zhang et al. [25], plantlets were treated with 200 mM of NaCl for 24h and then leaves were collected from stressed plants, plantlets treated with water were used as controls. Three independent biological replicates were performed for each experiment. All samples were frozen and stored in liquid nitrogen until use.

### 4.3. Library Construction and Sequencing for RNA-seq

In order to construct cDNA libraries, total RNAs were extracted from the control and the NaCl treated plant using Trizol RNA extraction kit (Life Technology, Beijing, China) according to the manufacturer’s instruction. Six samples were sequenced by Novogene (Tianjin, China) using Illumina HiSeq2500 system.

### 4.4. Transcriptome Assembly and Bioinformatics Analysis

Transcriptome assembly was accomplished based on the left.fq and right.fq using Trinity [17] with min_kmer_cov set to 2 by default and all other parameters set default. In brief, the left files (read1 files) from all libraries/samples were pooled into one big left.fq file, and right files (read2 files) into one big right.fq file. Gene function was annotated based on Nr (NCBI non-redundant protein sequences), Nt (NCBI non-redundant nucleotide sequences), Pfam (Protein family), KOG/COG (Clusters of Orthologous Groups of proteins), Swiss-Prot (A manually annotated and reviewed protein sequence database), KO (KEGG Ortholog database), GO (Gene Ontology) databases. Gene expression levels were estimated by RSEM [41] for each sample. Clean data were mapped back onto the assembled transcriptome. Read counts for each gene were obtained from the mapping results. For differential expression analysis, prior to differential gene expression analysis, for each sequenced library, the read counts were adjusted by edgeR program package [42] through one scaling normalized factor. Differential expression analysis of six samples was performed using the DEGseq R package [43]. 

All transcripts were searched against the latest versions (as of August 2018) of Nr (nonredundant) database (http://www.ncbi.nlm.nih.gov/) and the Swiss-Prot database (http://www.gpmaw.com/html/swiss-prot. html) using the BLAST program with an *e* < 10^−5^. The transcripts with the top hits were selected as unigenes. Open reading frames (ORFs) were predicted using the GetORF program contained in the EMBOSS software package. The Blast2GO program was used for GO annotation (http://www.geneontology.org), and the unigenes were aligned to the eggNOG (evolutionary genealogy of genes: non-supervised orthologous groups) database (http://www.ncbi.nlm.nih.gov/COG/) to identify functional categories. The KEGG database (http://www.genome.jp/kegg/) was used for pathway annotation. All searches were conducted using an e-value cut-off of 10^−5^. GO terms were downloaded from the GO Analysis Toolkit and Database for Agriculture Community (AGRI go, http://bioinfo.cau.edu.cn/agriGO/download.php). All the genes identified with significant differential expression (*p* < 0.05) and FC >2 in this study were used as inputs to carry out GO enrichment analysis. Gene Ontology (GO) enrichment analysis of the differentially expressed genes (DEGs) was implemented by the GOseq R packages based Wallenius non-central hyper-geometric distribution [44] that can adjust for gene length bias in DEGs. KEGG pathway enrichment analysis used KOBAS [19] software to test the statistical enrichment of the differential expression genes in the KEGG pathways. In the scatterplot, the rich factor is the ratio of the differentially expressed gene number to the total gene number in a certain pathway.

### 4.5. Quantitative RT-PCR

The candidate DEGs in response to salt stress were selected to validate the reliability of the RNA-seq data using quantitative RT-PCR following the previously reported procedures [45,46]. Gene-specific primers were listed in Table S8. *BhActin* was used as a reference gene. Three independent biological replicates were performed. The results from gene-specific amplification were analyzed using the comparative *Cq* method, which uses an arithmetic formula, 2^−ΔΔ*Cq*^, to achieve results for relative quantification [47]. *Cq* represents the threshold cycle.

## 5. Conclusions

We sequenced and comparatively analyzed the transcriptomes from the leaves of wild type *B. halophila* plants and plants with salt treatment. This work enabled us to characterize gene expression profiles and identify functional genes related to salt tolerance. A total of 519 genes were differentially expressed under salt stress. These DEGs appear to be involved in many aspects, such as the SOS pathway, ion transport and uptake, antioxidant enzyme, ABA signal pathway and so on. It has been shown that one gene encoding the AT-Hook Motif Nuclear Localized transcription factor and three genes encoding dehydrins, might play important roles in response to salt stress in *B. halophila*. The results provide good candidate genes to breed salt tolerant plants, and will be helpful in understanding of the molecular mechanisms of salt stress in *B. halophila*.

## Figures and Tables

**Table 1:** Summary of the sequencing data of the Betula halophila transcriptome.

Sample	Raw Reads	Clean Reads	Clean Bases	Error (%)	Q20 (%)	Q30 (%)	GC Content(%)
CK_1	68,348,352	66,834,236	10.03G	0.03	96.5	94.17	46.88
CK_2	75,684,144	73,359,338	11G	0.03	96.48	94.14	47.4
CK_3	70,510,750	69,082,978	10.36G	0.03	96.25	93.83	47.29
SC_1	86,101,536	84,155,542	12.62G	0.03	96.54	94.23	47.28
SC_2	77,402,824	75,577,004	11.34G	0.03	96.49	94.16	47.33
SC_3	84,837,142	82,931,756	12.44G	0.03	96.45	94.12	47.29

**Table 2:** Summary of function annotation of the *Betula halophila* transcriptome.

	Number of Unigenes	Percentage (%)
Annotated in NR	51,105	43.64
Annotated in NT	45,933	39.22
Annotated in KO	18,876	16.12
Annotated in SwissProt	40,624	34.69
Annotated in PFAM	40,661	34.72
Annotated in GO	41,116	35.11
Annotated in KOG	15,572	13.29
Annotated in all Databases	8973	7.66
Annotated in at least one Database	64,551	55.12
Total Unigenes	117,091	100

## Supplementary Materials

Supplementary materials can be found at http://www.mdpi.com/1422-0067/19/11/3412/s1. The sequencing data have been submitted to the SRA database under the accession number SRP146369.

## Abbreviations

AHL	AT-Hook Motif Nuclear Localized gene
BLAST	the Basic Local Alignment Search Tool
DEGs	differentially expressed genes
DHNs	dehydrins
GO	Gene ontology
KEGG	Kyoto Encyclopedia of Genes and Genomes
KOG	Eukaryotic Orthologous Groups
LEA	Late Embryogenesis Abundant
Nr	NCBI non-redundant protein sequence database
Nt	NCBI nucleotide sequences
ORFs	Open reading frames
Pfam	Protein family
SOS	Salt Overly Sensitive
SwissProt	Swiss-prot sequence data bases
