# Supplementary material for: Transcriptomic Analysis of Betula halophila in Response to Salt Stress
Source: Int J Mol Sci. 2018 Oct 31;19(11):3412. doi: 10.3390/ijms19113412 (PMC6274945; doi:10.3390/ijms19113412)
Supplement: Supplementary file 1 [file ijms-19-03412-s001.zip › Supplementary File for proof/Supplementary Figure 1.pdf]

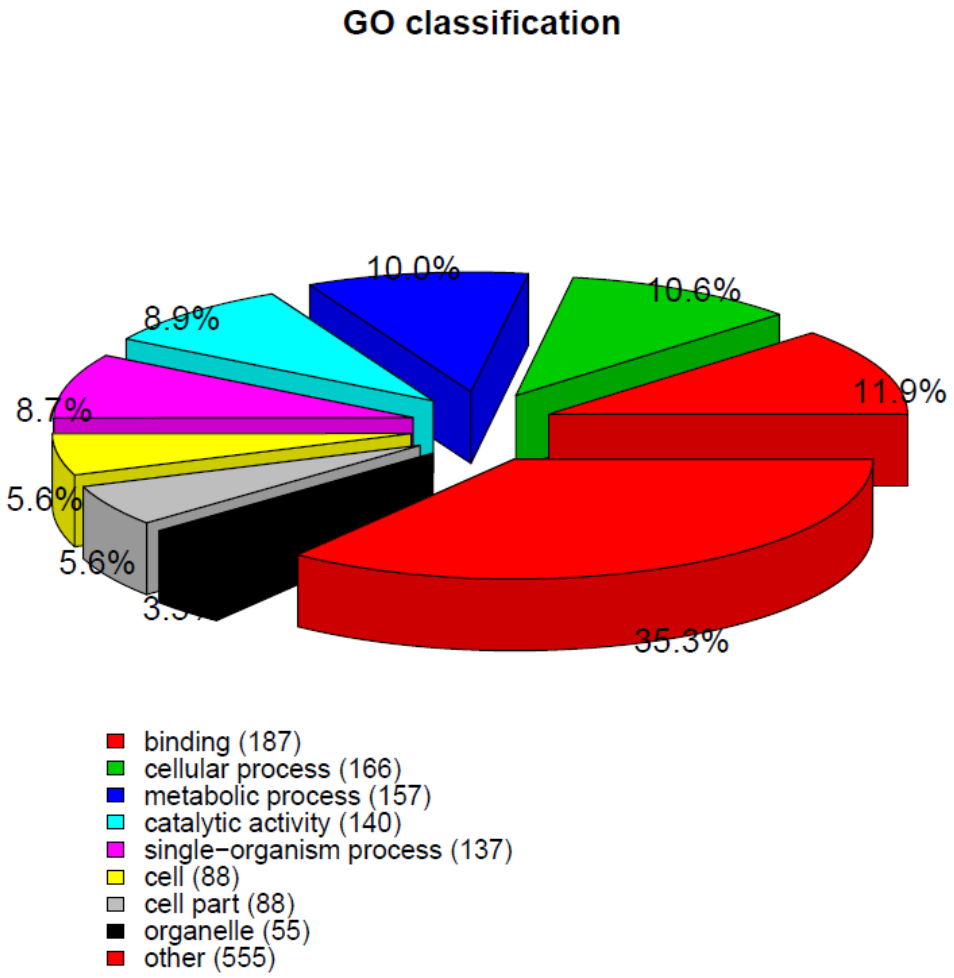
GO classification
10.0%
10.6%
8.9%
11.9%
8.7%
5.6%
5.6%
35.3%
binding (187)
cellular process (166)
metabolic process (157)
catalytic activity (140)
single−organism process (137)
cell (88)
cell part (88)
organelle (55)
other (555)

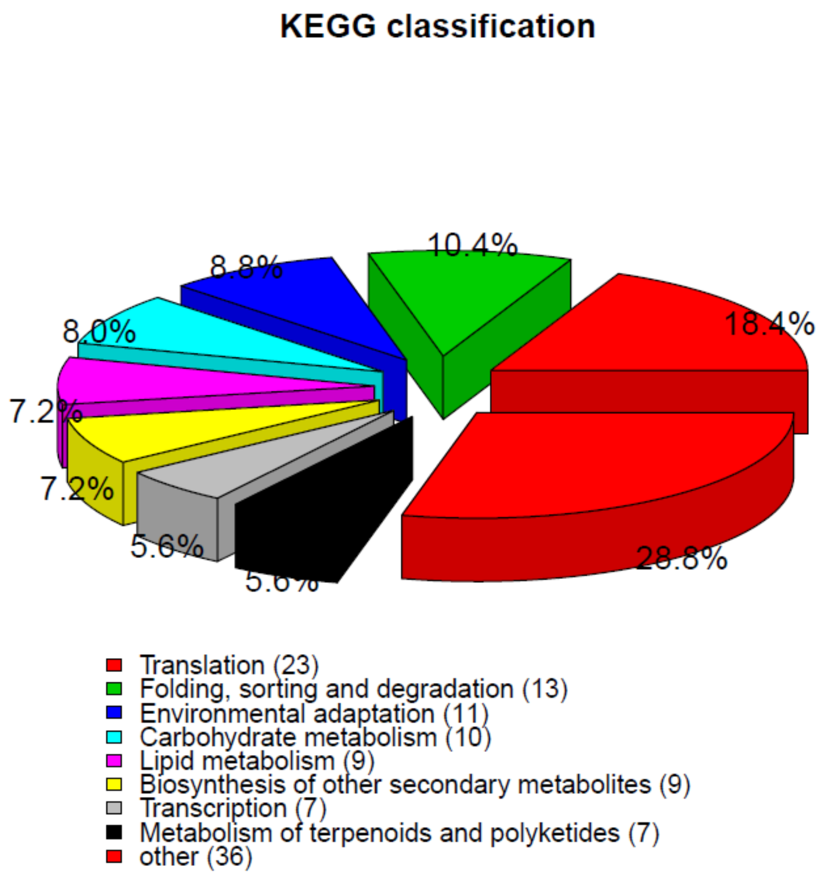
KEGG classification
10.4%
8.8%
18.4%
8.0%
7.2%
7.2%
5.6%
5.6%
28.8%
Translation (23)
Folding, sorting and degradation (13)
Environmental adaptation (11)
Carbohydrate metabolism (10)
Lipid metabolism (9)
Biosynthesis of other secondary metabolites (9)
Transcription (7)
Metabolism of terpenoids and polyketides (7)
other (36)
